# How Gameful Experience Affects Public Knowledge, Attitudes, and Practices Regarding COVID-19 Among the Taiwanese Public: Cross-sectional Study

**DOI:** 10.2196/26216

**Published:** 2021-04-06

**Authors:** Li-Hsun Peng, Ming-Han Bai

**Affiliations:** 1 Department of Creative Design National Yunlin University of Science and Technology Douliou, Yunlin Taiwan; 2 Graduate School of Design National Yunlin University of Science and Technology Douliou, Yunlin Taiwan

**Keywords:** COVID-19, knowledge, attitude, practice, serious game, gameful experience

## Abstract

**Background:**

In 2019, with the COVID-19 pandemic sweeping across the globe, public health systems worldwide faced severe challenges. Amid the pandemic, one simulation game, *Plague Inc.,* has received substantial attention. This game has indirectly drawn greater public attention to public health issues by simulating pathogen transmission and disease symptoms.

**Objective:**

Against this backdrop, this research investigates whether the gameful experience of *Plague Inc.* has indirectly affected public knowledge, attitudes, and practices (KAP) regarding COVID-19.

**Methods:**

An online survey was conducted through social networking services in Taiwan from May 6-28, 2020.

**Results:**

A total of 486 subjects participated in this study, of which 276 (56.8%) had played *Plague Inc.* This study had several findings. First, participants who had played *Plague Inc.* demonstrated higher levels of knowledge (*P*=.03, median 7, IQR 7-8) and attitudes (*P*=.007, median 8, IQR 7-8) than participants who had not played *Plague Inc.* (knowledge: median 7, IQR 6-8; attitude: median 7, IQR 6-8). Second, there was a significant correlation between creative thinking (ρ=.127, *P*=.04) and dominance (ρ=.122, *P*=.04) in attitude. Finally, there was a significant correlation between creative thinking (ρ=.126, *P*<.001) and dominance (ρ=.119, *P*=.049) in practice.

**Conclusions:**

Serious games highlighting the theme of pathogen transmission may enhance public knowledge and attitudes regarding COVID-19. Furthermore, the creative thinking and dominance involved in gameful experiences may act as critical factors in public attitudes and practices regarding COVID-19. These findings should be further verified through experimental research in the future.

## Introduction

At the end of 2019, an outbreak of pneumonia of unknown cause was detected in Wuhan, China, and it was quickly discovered to be caused by a novel coronavirus, SARS-CoV-2 [1]. On January 21, 2020, Taiwan reported the first confirmed COVID-19 case overseas [2]. As of March 21, 2021, more than 270 million diagnosed cases and 1 million deaths had been reported globally [3]. Although vaccines are considered one of the means of prevention of infectious diseases such as COVID-19, it takes time to verify their safety before they can be brought to market [4].

In the face of the severe threat of COVID-19, implementing controls on health care services and communities has been deemed an effective strategy [5]. In addition, a general population with higher levels of knowledge regarding pathogens is considered more likely to adopt the correct preventive measures and thereby reduce the prevalence rate of infections [6,7]. Therefore, many governments in the world have proactively provided the latest valid COVID-19 information and guidelines on epidemic prevention to their citizens using mainstream media or social media [8].

Promoting public attention toward health and medical care using game feedback has been proposed, as it helps the public understand possible threats to health and medical challenges when facing a difficult situation related to public health and safety [9]. The Centers for Disease Control and Prevention in the United States and the University of Derby in the United Kingdom have also attempted to impart knowledge about virus transmission and microbiology using educational games [10,11].

Teaching medical knowledge through the form of games has been found to be as effective as traditional text-based teaching methods [12]. In addition to teaching accurate information in a guided and context-based manner, incorporating knowledge pertaining to food and drug safety into video games has been proven to effectively raise the level of relevant knowledge among youngsters [13,14]. Moreover, teaching cardiopulmonary resuscitation through serious games has also been shown to result in a higher retention rate of knowledge and skills [15,16]. Teaching hand hygiene and diarrhea prevention methods through educational games has been confirmed to enhance users’ awareness and practice of public health habits [17,18]. Although serious games have potential for use in medical education and promote proactiveness in learners, it is necessary to strike a balance between instructional theories, educational content, and game interaction to effectively enhance learning performance [19].

**Figure 1:**
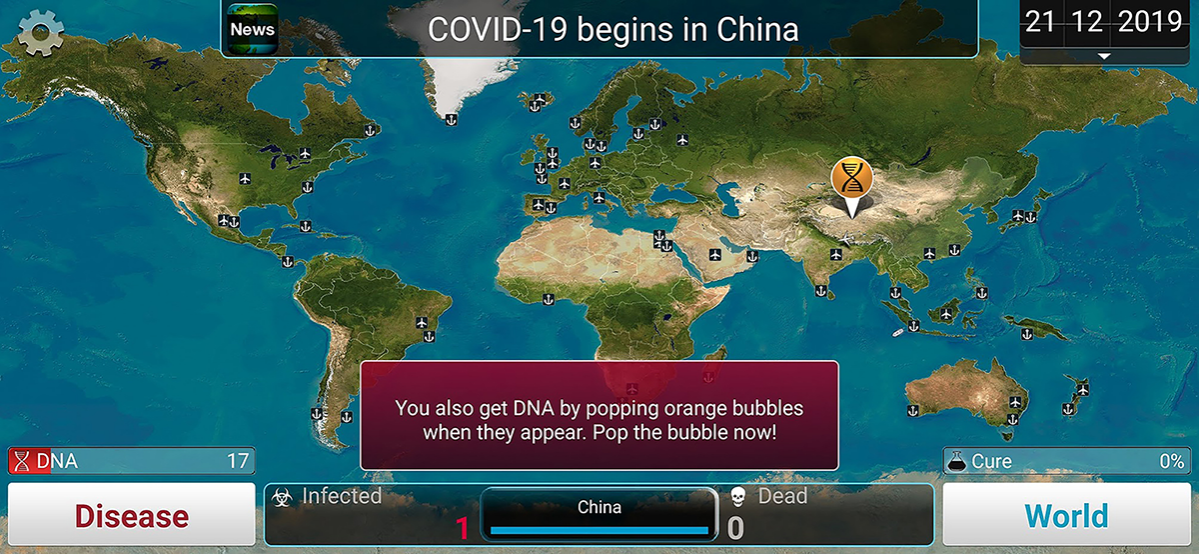
Game guild levels of Plague Inc.

*Plague Inc.* is a pathogen simulation game that attracts widespread public attention whenever a pandemic breaks out. This game introduces the public to epidemiology in an unconventional way, allowing users to obtain knowledge of the transmission and characteristics of pathogens through gaming [20]. As a form of new media, *Plague Inc.* employs simple graphics but has diverse charts and functions (Figure 1) [21]. The public may acquire scientific knowledge through intuitive simulation games. In addition, students may also learn about and analyze pathogen transmission and disease symptoms through gameful experiences [22,23].

However, the behaviors that players exhibit while playing games are not just intended for entertainment and recreation. Players’ personality traits are believed to be projected onto their behavior and choices during a game [24,25]. In particular, educational issue–based games not only integrate common gaming elements such as points, badges, and rankings into their design but also offer experiences that enhance extrinsic and intrinsic motivations for players [26-29]. Gamification technology has been found to have a positive effect on health and well-being [30]. Moreover, it has also been deemed to have an important mediating role in health behaviors [31]. Gamification technology has proven to be an effective tool for clinical nursing skill training. However, users’ feelings and attitudes toward the gamification content should be considered [32]. In addition, both gamification technologies and gameful experience can result in psychological or behavioral changes [33].

### Objective

This research aims to investigate and explore the effects of gameful experience of *Plague Inc.* on public knowledge, attitudes, and practices (KAP) regarding COVID-19 through online surveys.

## Methods

### Study Design

This research used a cross-sectional study design to explore the impact of Taiwanese gameful experiences on KAP regarding COVID-19.

From May 6-28, 2020, we collected data through an online survey, the link to which was published on social networking services (such as Facebook, PTT Bulletin Board System, internet game forums).

The target population was residents of Taiwan, the population of which was 23,591,920 [34] in April 2020. We used the sample size calculator provided by Rao Soft Inc and selected a 95% confidence level, 5% margin of error, and 23,591,920 population size; the calculated recommended sample size was 385.

This study was conducted in line with the Declaration of Helsinki. All participants were anonymous and provided informed consent. They clearly understood the research content in the online questionnaire [35]. The survey information sheet contained an introduction, background, goals, anonymity statement, and questionnaire descriptions. The average survey completion time was 5 minutes.

### Survey Instrument

The online survey consisted of four parts: demographics, KAP of COVID-19, game experience of *Plague Inc.*, and a gameful experience scale.

The first part includes demographic variables such as gender, age range, education, living arrangement, and occupation.

The second part pertains to the revised COVID-19 scale. The scale contains 27 questions on the three dimensions of COVID-19—namely, knowledge, attitudes, and practices. Each dimension has 9 questions, with 0 as the lowest score and 9 as the highest. Respondents are required to select “Yes,” “No,” or “Uncertain” for each question. For each correctly answered question, one point is added to the total score of the dimension that contains the question. Moreover, no point was awarded for questions with an incorrect or “Uncertain” answer.

The original scale was used to discuss the KAP regarding Middle East respiratory syndrome coronavirus (MERS-CoV) during the annual Hajj pilgrimage [36], and it was adjusted and translated to Chinese. In addition, five experts in clinical medical treatment and public health were invited to examine the questionnaire content to ensure the questionnaire’s validity. The questionnaire is shown in Table 1.

**Figure 2:**
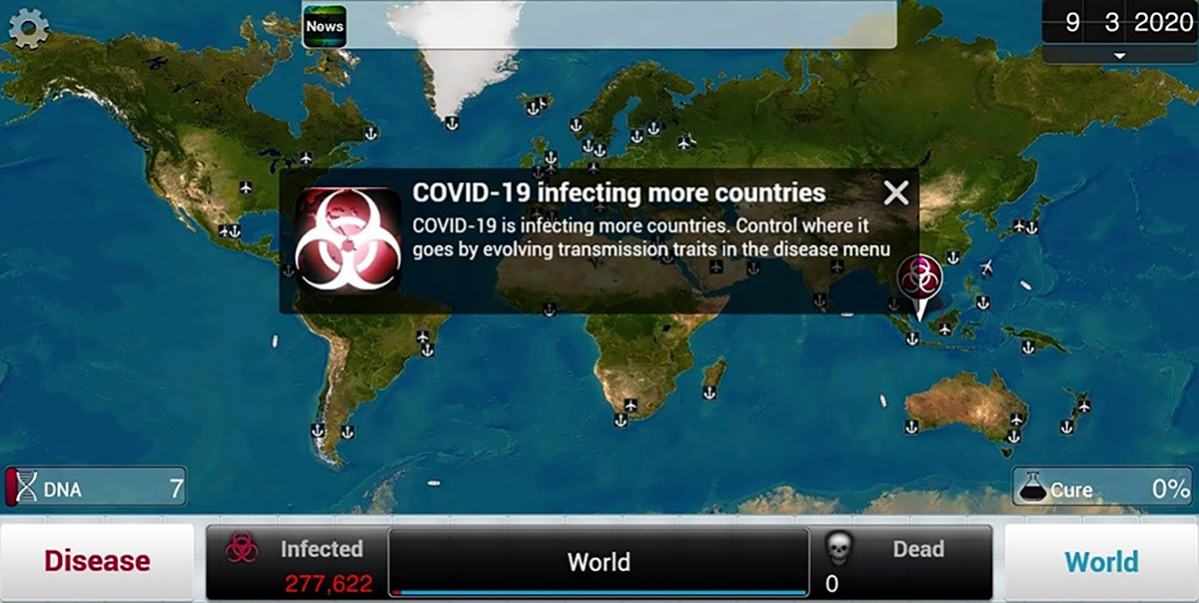
Screenshot of *Plague Inc.*

The third part is about gameful experience of *Plague Inc.,* including “ever played *Plague Inc.*,” “time since last played,” and “total playtime ranges.” In this part, screenshots were used to describe the game’s content (Figure 2).

The fourth part is the gameful experience scale consisting of 27 questions, divided into 6 dimensions: enjoyment, absorption, creative thinking, activation, absence of negative affect, and dominance [37]. The scale is a 5-point Likert scale, with options from 1 (strongly disagree) to 5 (strongly agree).

This scale was also used in nursing-related research and was considered to demonstrate excellent reliability and validity. It can be used to evaluate nursing students’ gaming experiences during training [38]. Three practitioners from the gaming industry were invited to give suggestions on modifying the questionnaire after translation.

**Table 1:** Questionnaire of knowledge (K), attitudes (A), and practices (P) regarding COVID-19.

Questions	Correct response (%)
K1. The incubation period of COVID-19 is from 2 to 14 days.	81.3
K2. Stomach cramps, loss of appetite, nausea, and vomiting are the main symptoms of COVID-19.	38.9
K3. People with comorbidities are more likely to be infected.	77.0
K4. COVID-19 spreads through close contact with an infected person, such as caring for and/or living with them.	94.7
K5. The primary source of COVID-19 is a plant.	89.1
K6. Antibiotics are the first line of treatment.	52.5
K7. Vaccinations for COVID-19 are available.	91.4
K8. COVID-19 can be fatal.	96.9
K9. Isolation of patients with COVID-19 is essential to ensure effective implementation of infection control measures.	99.0
A1. Do you agree that there is no risk of contracting COVID-19 when going out and traveling?	83.7
A2. Do you agree that taking antibiotics can treat COVID-19?	48.8
A3. Do you agree that pain medication and/or fever medications cannot relieve COVID-19 symptoms?	38.7
A4. Do you agree that washing your hands with soap and water for at least 30 seconds can prevent disease transmission?	95.7
A5. Do you agree that avoiding undercooked meat or food prepared under unsanitary conditions can prevent the transmission of disease?	87.4
A6. Do you agree that avoiding contact with live animals such as bats can prevent the spread of disease?	86.6
A7. Do you agree that avoiding contact with ill people can prevent the spread of disease?	97.3
A8. Do you agree that before attending public gatherings or traveling, one must have the necessary information about COVID-19?	95.9
A9. Do you agree that reporting COVID-19 symptoms to local health authorities is essential to prevent further disease transmission?	98.8
P1. At a public gathering, did you cover your mouth when sneezing/coughing?	97.3
P2. At a public gathering, did you wash your hands with soap and water after sneezing/coughing?	59.5
P3. At a public gathering, did you wash your hands before preparing or eating foods?	80.7
P4. At a public gathering, did you wash your hands after contact with possibly contaminated surfaces or materials?	85.0
P5. At a public gathering, did you avoid close contact with people when you were sick?	91.6
P6. At a public gathering, did you wear a face mask in heavily crowded areas?	96.7
P7. At a public gathering, did you share cups or eating utensils with other people?	78.8
P8. Did you consult a health care worker during a public gathering if you had a fever, cough, or difficulty breathing?	92.4
P9. At a public gathering, did you avoid direct hand contact with your eyes, nose, and mouth?	78.0

### Game

*Plague Inc.* is a strategic simulation game that simulates how various pathogens are spread throughout the world. It was released on the App Store and Google Play in 2012. In 2014, *Plague Inc: Evolved* was launched on Steam, a platform for PC games.

At the beginning of each game, players can repeatedly spread new pathogens to different countries. Using DNA points in the game, players try to achieve the goal of eliminating all of mankind by employing different modes of transmission, symptoms, and abilities, and influencing the infectivity, severity, and lethality of the viruses.

**Figure 3:**
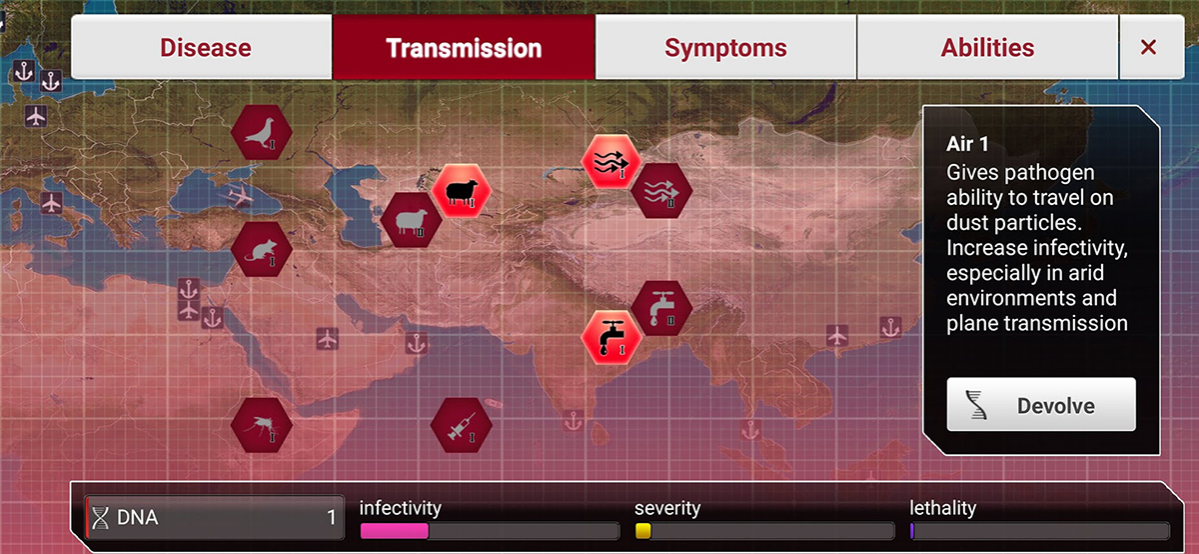
Screenshot of Plague Inc.: disease transmission.

Players may indirectly control the speed at which infectious pathogens spread by selecting different routes of transmission, as shown in Figure 3. In particular, the game simulates how pathogens spread via airplanes and boats, increasing the number of infected countries and individuals.

**Figure 4:**
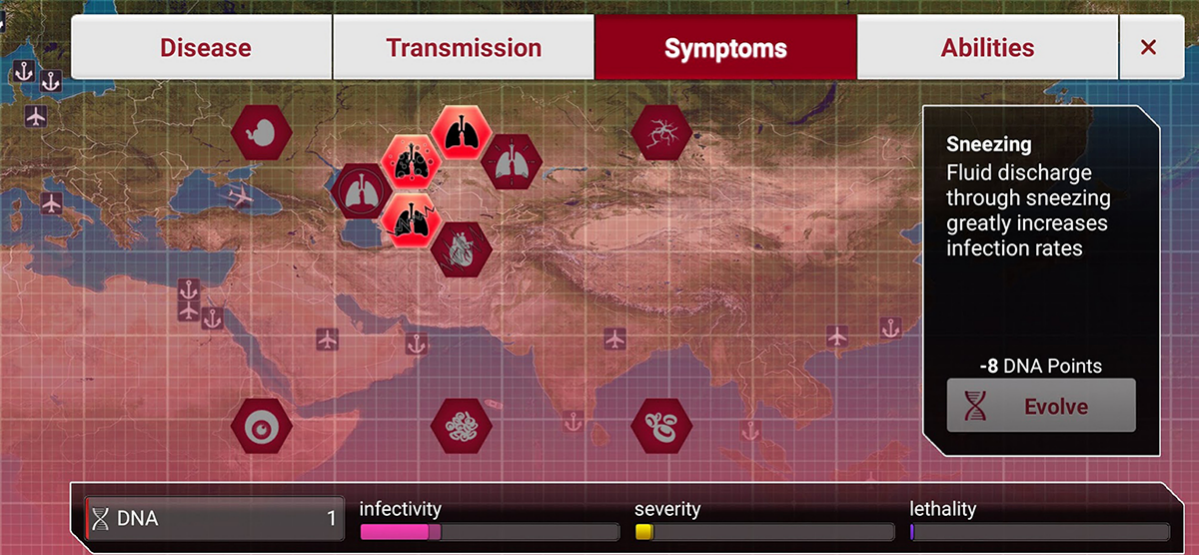
Screenshot of Plague Inc.: disease symptoms.

Players may change the infectivity and lethality of pathogens by selecting different symptoms, as shown in Figure 4. They may keep the death toll from ascending too rapidly by balancing infectivity and lethality, while allowing pathogens to continue spreading through infected individuals.

**Figure 5:**
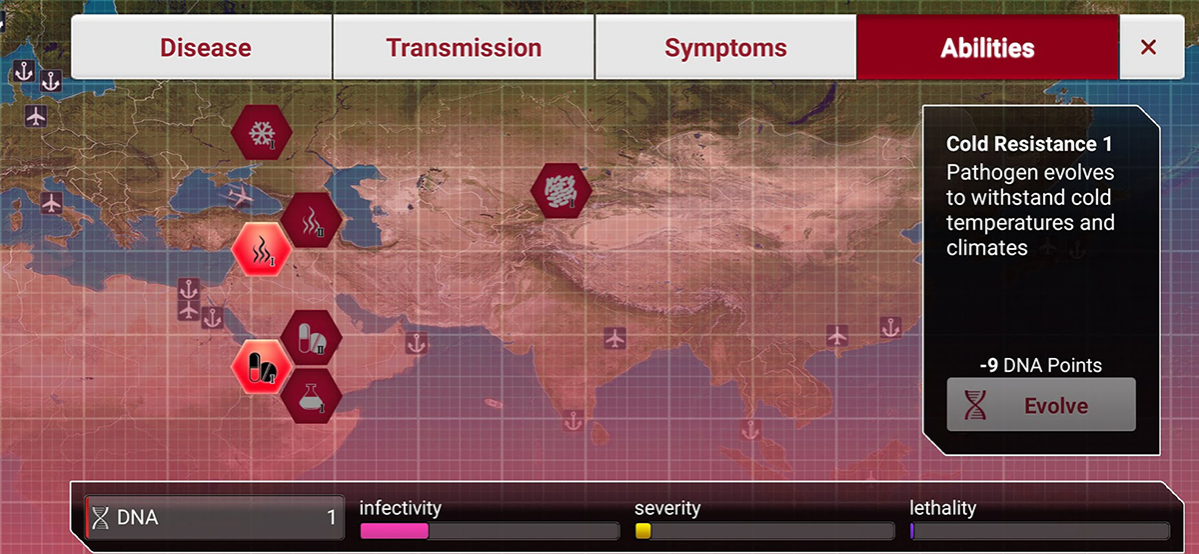
Screenshot of Plague Inc.: disease abilities.

*Plague Inc.* simulates countries with different climates and latitudes. In addition, as the game progresses, vaccines will appear to prevent players from winning. Therefore, players have to use their abilities to influence the pathogen’s temperature and drug resistance, as shown in Figure 5.

### Data Analysis

Based on the normality test, this study used nonparametric statistics, the Mann–Whitney *U* test, and the Kruskal–Wallis test to verify the KAP of COVID-19 to understand whether the KAP of COVID-19 was affected by gaming experience. In addition, we used the chi-square test to analyze whether there is a significant difference between whether participants have played *Plague Inc.* and their demographic characteristics and the Spearman rank correlation test to analyze the correlation between gameful experience and COVID-19 KAP.

In this study, the Cronbach α test was used to detect the internal consistency of the KAP of COVID-19 questionnaire and the gameful experience scale. For the KAP of COVID-19 questionnaire, Cronbach α=.82, while Cronbach α=.994 for the gameful experience scale; the results had acceptable internal consistency [39].

## Results

A total of 486 participants were recruited for this study; demographic characteristics are shown in Table 2. Participants included 200 (41.2%) women and 286 (58.8%) men. The majority of participants were aged 20-29 years (n=323, 66.5%), had a university degree (n=305, 62.8%), were living with others (n=365, 75.1%), and were part of the labor force (n=335, 68.9%).

In total, 276 (56.8%) participants indicated they have played *Plague Inc.* and 210 (43.2%) have never played the game (Table 3).

**Table 2:** Demographic characteristics (N=486).

Variables			Participants, n (%)
**Gender**			
	Male	286 (58.8)	
	Female	200 (41.2)	
**Age group (years)**			
	20-29	323 (66.5)	
	30-39	127 (26.1)	
	40-49	32 (6.6)	
	≥50	4 (0.8)	
**Education**			
	High school or below	20 (4.1)	
	University	305 (62.8)	
	Graduate school	161 (33.1)	
**Living arrangement**			
	Alone	121 (24.9)	
	With others	365 (75.1)	
**Occupation**			
	Labor force	335 (68.9)	
	Non–labor force	103 (21.2)	
	Unemployed	48 (9.9)	

**Table 3:** Descriptive statistics regarding participants’ game experience (N=486).

Variables			Participants, n (%)
**Ever played**			
	Yes	276 (56.8)	
	No	210 (43.2)	
**Time since last played**			
	<1 week	26 (5.3)	
	<1 month	63 (13)	
	<6 months	83 (17.1)	
	<1 year	25 (5.1)	
	≥1 year	79 (16.3)	
**Total playtime**			
	<1 hour	63 (13)	
	1-10 hours	127 (26.1)	
	≥10 hours	86 (17.7)	

The chi-square test was used to examine whether there is a significant difference in the demographic characteristics of participants who have played *Plague Inc.* and those who have not. The analysis results are shown in Table 4.

The analysis results show significant differences in gender (*P*<.001) and age (*P*=.006) between participants who have and have not played *Plague Inc.* However, no significant difference is observed in education, living arrangement, or occupation.

In terms of gender, males who have played *Plague Inc.* account for a higher percentage (196/286, 68.5%) than those who have not (90/286, 31.5%), whereas more females have not played *Plague Inc.* (never played: 120/200, 60%; ever played: 80/200, 40%).

As for the different age groups, among participants aged 20-29 years, more have played *Plague Inc.* (195/323, 60.4%) than not (128/323, 39.6%); among those aged 30-39 years, more have played (69/127, 54.3%) than not (58/127, 45.7%); among those aged 40-49 years, more have not played (20/32, 62.5%) than have played (12/32, 37.5%); and finally, among participants aged ≥50 years, none have played Plague Inc. before (4/4, 100%).

**Table 4:** Chi-square test for demographic characteristics between those who have or have not played Plague Inc. (N=486).

Variables		Participants, n	Ever played *Plague Inc.,* n (%)		Chi-square test			
			Yes (n=276)	No (n=210)	*χ*^2^ (*df*)		*P* value	
**Gender**						39 (1)		<.001
	Male	286	196 (68.5)	90 (31.5)				
	Female	200	80 (40)	120 (60)				
**Age group (years**)						12.1 (3)		.006
	20-29	323	195 (60.4)	128 (39.6)				
	30-39	127	69 (54.3)	58 (45.7)				
	40-49	32	12 (37.5)	20 (62.5)				
	≥50	4	0 (0)	4 (100)				
**Education**						1.2 (2)		.54
	High school or below	20	11 (55)	9 (45)				
	University	305	179 (58.7)	126 (41.3)				
	Graduate school	161	86 (53.4)	75 (46.6)				
**Living arrangement**						1.8 (1)		.18
	Alone	121	75 (62)	46 (38)				
	With others	365	201 (55.1)	164 (44.9)				
**Occupation**						3 (2)		.22
	Labor force	335	197 (58.8)	138 (41.2)				
	Non–labor force	103	57 (55.3)	46 (44.7)				
	Unemployed	48	22 (45.8)	26 (54.2)				

The Mann-Whitney *U* and Kruskal-Wallis tests were used to assess any differences in KAP of COVID-19 between participants who had ever or never played *Plague Inc.,* while the Kruskal-Wallis test was used to determine whether differences in KAP of COVID-19 were related to differences in time since last played and total playtime, as shown in Table 5.

There was a statistically significant difference between knowledge and attitude regarding COVID-19 for the item of whether a participant had ever played the game *Plague Inc.*

The scores on questions related to knowledge of COVID-19 were lower among never-played participants (median 7, IQR 6-8) than among ever-played participants (median 7, IQR 7-8).

The scores on the questions about attitude regarding COVID-19 were lower among never-played participants (median 7, IQR 6-8) than among ever-played participants (median 8, IQR 7-8). The results indicated that the time since last played had a significant effect for the item of attitude regarding COVID-19. The scores on the questions about attitude regarding COVID-19 were lower among never-played participants (median 7, IQR 6-8) than among those who had not played in over one year (median 8, IQR 7-9).

The results indicated that there was a significant difference in scores on the questions about attitude regarding COVID-19 among participants with different total playtimes. The scores on questions about attitude regarding COVID-19 were lower among never-played participants (median 7, IQR 6-8) than among those with a total playtime of 1-10 hours (median 8, IQR 7-8).

Spearman rank correlation was used to examine the relationship between KAP of COVID-19 and gameful experience (Table 6). The results showed that there was a significant correlation between creative thinking (ρ=.127, *P*=.04) and dominance (ρ=.122, *P*=.04) in attitude; in addition, there was a significant correlation between creative thinking (ρ=.126, *P*<.001) and dominance (ρ=.119, *P*=.049) in practice.

**Table 5:** Mann-Whitney U test and Kruskal-Wallis test for determining the effect of game experience on knowledge, attitudes, and practices regarding COVID-19 (N=276).

Variables		Knowledge score				Attitude score				Practice score		
		Median (IQR)	Mean (SD)	*P* value	Median (IQR)		Mean (SD)	*P* value	Median (IQR)		Mean (SD)	*P* value
**Ever played**												
	Yes	7 (7-8)	7.31 (1.23)	.03	8 (7-8)		7.46 (1.15)	.007	8 (7-9)		7.54 (1.51)	.39
	No	7 (6-8)	7.07 (1.2)		7 (6-8)		7.16 (1.27)		8 (7-9)		7.67 (1.46)	
**Last played**												
	<1 week	7.5 (7-8)	7.58 (0.99)	.26	8 (7-8)		7.62 (0.94)	.02	8.5 (7-9)		8.12 (1.11)	.18
	<1 month	7 (7-8)	7.3 (1.24)		7 (7-8)		7.32 (1.13)		8 (7-9)		7.75 (1.43)	
	<6 months	7 (6-8)	7.25 (1.4)		8 (7-8)		7.4 (1.24)		8 (6-9)		7.48 (1.48)	
	<1 year	8 (7-8)	7.4 (1)		7 (6-8)		7.12 (1.24)		8 (6.5-9)		7.4 (1.71)	
	≥1 year	7 (6-8)	7.25 (1.16)		8 (7-9)		7.68 (1.08)		8 (6-9)		7.3 (1.62)	
**Total playtime**												
	<1 hour	7 (6-8)	7.27 (1.31)	.10	7 (7-8)		7.32 (1.1)	.02	8 (7-9)		7.57 (1.55)	.47
	1-10 hours	7 (6-8)	7.23 (1.31)		8 (7-8)		7.53 (1.23)		8 (7-9)		7.63 (1.49)	
	≥10 hours	8 (7-8)	7.45 (1.01)		7 (7-8)		7.45 (1.06)		8 (6.75-9)		7.4 (1.51)	

**Table 6:** Spearman rank correlation of gameful experience with knowledge, attitudes, and practices regarding COVID-19 (N=324).

Variables	1	2	3	4	5	6	7	8	9
1. Knowledge	—^a^	—	—	—	—	—	—	—	—
2. Attitude	.458^b^	—	—	—	—	—	—	—	—
3. Practice	.087	.166^b^	—	—	—	—	—	—	—
4. Enjoyment	.020	.034	.049	—	—	—	—	—	—
5. Absorption	–.028	–.082	.031	.115	—	—	—	—	—
6. Creative thinking	.029	.127^c^	.126^c^	.361^b^	.351^b^	—	—	—	—
7. Activation	.076	.031	.052	.259^b^	.508^b^	.396^b^	—	—	—
8. Absence of negative affect	.067	–.087	–.115	–.279^b^	.012	–.147^c^	.087	—	—
9. Dominance	.003	.122^c^	.119^c^	.242^b^	.422^b^	.478^b^	.453^b^	–.141^c^	—

^a^Not applicable.

^b^*P*<.01.

^c^*P*<.05.

## Discussion

### Principal Findings

This study determined the demographics of members of the public who have played *Plague Inc.* The results show that the research participants who have played *Plague Inc.* are mainly males aged 20-39 years. Studies in Spain and Turkey also found that gender indirectly affects game type preferences [40,41]. Research in the United States discovered that although the majority of middle-aged and older adults do not play video games, age does not affect their preference for strategic simulation games [42].

Participants who have played *Plague Inc.* displayed higher levels of knowledge and attitudes toward COVID-19. Health education implemented through gameful experience has been proven to effectively enhance knowledge of leptospirosis [43]. In addition, integrating gamified media into medical education courses has been shown to effectively improve knowledge and skill performance among nursing students [44-46]. Moreover, designing applications to integrate with gameful experience to explore learning performance in professional nursing education has also been proven to generate a higher level of knowledge when compared to traditional teaching methods [15].

In particular, research participants who had played *Plague Inc.* one year before the outbreak of the COVID-19 pandemic had higher attitude scores for COVID-19 than those who had not. Designing medical clinical instructional content as a serious game and incorporating gameful experience into training may effectively enhance medical knowledge and confidence among nursing students. Moreover, students may repeatedly practice in the simulated environment, and thereby develop a self-guided learning strategy [47,48]. China’s research on COVID-19–related serious games also confirmed that educational games result in better performance in learning retention than online lectures [49].

Participants who have played *Plague Inc.* indicated higher creative thinking and dominance during their gameful experience. Their attitudes and practices regarding COVID-19 have a significant positive correlation. In addition, the creative thinking involved in gameful experience is considered to be one of the significant factors that affect learning performance [50]. Gamified learning materials have been proven to stimulate creative thinking in learners and improve their attitudes and soft skills for school subjects [51]. Moreover, simulated situations can also cultivate creative thinking and indirectly improve attitudes toward public health [52,53].

The game environment of *Plague Inc.* allows players to repeatedly experiment with different pathogens and features. During this process, players gain a better understanding of how to enhance the infectivity and lethality of a pathogen. The challenge experiences and strategies accumulated through playing the game may indirectly improve the players’ corresponding attitudes and practices toward pathogens. Previous research has demonstrated that the reason that effective learning performance is achieved through serious games is due to the concept of experience-based learning. In addition, with game situations designed for players to repeatedly try and challenge, such games may motivate players to reflect on the content and strategize accordingly [54].

### Limitations

This study has several limitations. First, the sample number may be insufficient to represent the general public of Taiwan. Second, as the survey was conducted on social networking websites, the surveys were mainly completed by males aged 20-39 years. This study lacks a broader scope of participants. Third, the research survey was conducted in May 2020; the COVID-19 pandemic was already easing in Taiwan by then. Moreover, the government and medical units had also implemented advocacy and educational messaging through multiple channels. As such, the public had developed a basic understanding of COVID-19. Fourth, as the game investigated in this study is a strategic simulation game, research participants may indirectly represent a specific group. Finally, *Plague Inc.* was not designed specifically for COVID-19. In addition, the goal of the game is to infect and kill all human beings. A new version of the game, *The Cure*, was launched on November 11, 2020, which allows players to promote antipandemic prevention and isolation measures by roleplaying as the World Health Organization; however, the public may still be unable to acquire precise knowledge of specific viruses via this game.

### Future Research

This research focuses on the relationship between public KAP regarding COVID-19 and the gameful experience of *Plague Inc.* However, there may be more factors that affect public KAP regarding COVID-19 other than the gameful experience of *Plague Inc.* Therefore, further research is required to explore possible interactions with other dimensions [31].

### Conclusions

This study has demonstrated that *Plague Inc.* provides pathogen simulation scenarios that allow players to continuously seek challenges. Members of the public who have played *Plague Inc.* exhibit higher levels of knowledge and attitudes regarding COVID-19. Moreover, the creative thinking and dominance developed in the gameful experience have been found to be correlated with attitudes and practices regarding COVID-19. Reinforcing creative thinking and dominance through serious games may be a valid approach. However, further verification is required.

Finally, it is imperative to heighten public awareness of the symptoms and transmission routes of COVID-19 amid the unprecedented crisis of the current pandemic. This study suggests that new media, such as games, should be employed to help the public accumulate learning experiences regarding pathogens and further establish public KAP toward pathogens. In this manner, the public may become more heedful of public health issues in the future.

## Abbreviations

KAP: knowledge, attitudes, and practices
MERS-CoV: Middle East respiratory syndrome coronavirus
